# Clinical significance of BPI-ANCA in patients with cystic fibrosis: a single center prospective study

**DOI:** 10.1038/s41598-023-45273-2

**Published:** 2023-10-24

**Authors:** Kenneth Iwuji, Adaobi Kanu, Stephanie Stroever, Kenneth Nugent, Abdul Hamood, Chris Scott, Stephany Navarro

**Affiliations:** 1 Division of Pulmonary and Critical Care Medicine, Department of Internal Medicine, Texas Tech University Health Sciences Center, Lubbock, TX USA; 2 Department of Pediatrics/Pulmonology, Texas Tech University Health Sciences Center, Lubbock, TX USA; 3 Clinical Research Institute, Texas Tech University Health Sciences Center, Lubbock, TX USA; 4 Department of Immunology & Molecular Microbiology, Texas Tech University Health Sciences Center, Lubbock, TX USA

**Keywords:** Genetics, Immunology

## Abstract

Recurrent pulmonary exacerbation due to infection and inflammation remain the major cause of mortality and morbidity in patients with cystic fibrosis (CF). Increased levels of BPI-ANCA have been linked to *Pseudomonas* colonization and pulmonary exacerbations in patients with CF. The majority of these studies were done in Europe, and it is unclear whether similar findings are true in CF patients who lives in United States. In our single center study of 47 patients with CF, the prevalence of BPI-ANCA was 19% at baseline and 15% at annual follow-up visit. Overall, there were no statistical differences noted in FEV1 and frequency of pulmonary exacerbations in CF patients who were positive for BPI-ANCA compared to those who were negative for BPI-ANCA. The role of BPI-ANCA in patients with CF still remains unclear.

## Introduction

Cystic fibrosis (CF) is a multisystem progressive autosomal recessive disease caused by a mutation in the cystic fibrosis transmembrane conductance regulator (CFTR) gene causing impairment of chloride transport. The majority of people with CF are Caucasians and are at least 18 years of age. About 40,000 people in the United States are living with CF, and more than 1000 new cases are diagnosed each year^1^. Despite being a multisystem disease, concurrent pulmonary infections (particularly with *Pseudomonas aeruginosa* and *Staphylococcus aureus*) and chronic airway inflammation remain the major cause of morbidity and mortality in these patients.

Bactericidal/permeability increasing (BPI) protein is found in azurophil granules of neutrophils. It has a potent antimicrobial activity against Gram-negative bacteria, such as *P. aeruginosa,* by contributing to its opsonization and neutralization of its endotoxins, thereby killing the bacteria^2^. Studies indicate that BPI is a common target for antineutrophil cytoplasmic autoantibodies (ANCA). These ANCA antibodies with BPI specificity (BPI-ANCA) have been identified in several inflammatory disorders and infectious diseases, such as CF, chronic obstructive lung disease, rheumatoid arthritis, vasculitis, and inflammatory bowel disease^3,4^. This BPI-ANCA complex prevents BPI-induced *P. aeruginosa* killing^4,5^. In a recent meta-analysis, the prevalence of BPI-ANCA in patients with CF varied from 17.9 to 83% with a pooled prevalence of nearly 50%^6^.

BPI-ANCA is found in the serum and airways of patients with CF and correlates with *P. aeruginosa* colonization and infections resulting in recurrent pulmonary exacerbations. Elevated BPI-ANCA levels are also associated with a poor prognosis^7–9^. The majority of the BPI-ANCA studies have been done outside of the United States, and it is unclear if the reported findings are replicable in patients with CF who live in the United States. The aim of this study was to characterize BPI-ANCA levels in CF patients living in the United States.

## Methods

### Patients

Of the 105 non-transplanted patients who are registered at the Cystic Fibrosis Care Center at Texas Tech University Medical Center in Lubbock, Texas, 47 subjects met the eligibility criteria and were included in the study after informed consent was signed. Patients with confirmed diagnosis of cystic fibrosis, age 5–50 years old, and receiving care at TTUHSC CF center were included in study enrollment. Patients with confounding factors (age below 5 years of age or above 50 years of age, current pregnancy or breastfeeding, currently institutionalized, history of organ transplant, on immunosuppressive agents, and hospital admission in the last 30 days) were excluded from enrollment. Characteristics of the patient enrolled in the study are listed on Table 1. The enrollment period lasted from June 2, 2018, to September 30, 2021. Five patients were excluded from the final results due to loss of follow-up or incomplete data (three patients relocated to another city and the other two had incomplete testing) (Table 2). This study was approved by the Texas Tech University Health Sciences Center Institutional Review Board, and written consent from the participants (or the legal guardian if the patient is a minor) was obtained prior to inclusion (IRB #: L18-136). This study was performed in accordance with the relevant guidelines and regulation. As part of their routine care, all participants had documented genetically confirmed CF diagnosis. The results of their mutation analysis and other pertinent clinical data were obtained from patients' records. Baseline IgG BPI-ANCA levels, lung function studies, medication history, and microbiology results were obtained at the time of enrollment. Repeat testing, including clinical outcomes (alive, number of exacerbations, spirometry result, lung transplant, and mortality), was performed 1 year after enrollment into the study. To confirm the validation of the BPI-ANCA assay, five healthy subjects were also enrolled and tested for BPI-ANCA levels.

**Table 1:** Baseline characteristics of patients with cystic fibrosis enrolled in a study to assess prevalence of BPI-ANCA in a West Texas CF clinic (N = 47).

Characteristic	Mean (SD)
Age at enrollment	18.8 (9.2)
Sex (male)	20 (42.6)
History of pancreatic insufficiency (yes)	45 (95.7)
History of asthma (yes)	29 (61.7)
History of gastroesophageal reflex disease (yes)	7 (14.9)
History of diabetes (yes)	10 (21.3)
Current smoker (yes)	1 (2.1)
*Pseudomonas aeruginosa* colonization (yes)	12 (25.5)

SD: standard deviation; IQR: interquartile range; N: number; %: percentage.

**Table 2:** Clinical characteristics of patients with cystic fibrosis in a West Texas CF clinic at baseline and follow-up 1 year after enrollment.

Characteristic		Baseline (N = 47)	Follow-up (N = 42)
FEV1% predicted^a^	[median (IQR)]	77 (55, 86)	81 (71, 89)
Pulmonary exacerbations^b^	[median (IQR)]	3 (2, 4)	4 (3, 5)
Anti-BPI IgG^c^	[median (IQR)]	4.2 (3.0, 7.4)	3.6 (2.7, 6.9)
BPI-ANCA positive^d^	[N (%)]	9 (19.2)	6 (15.4)
Antibiotic use	[N (%)]	34 (72.3)	34 (80.1)
CFTR modulator use^e^	[N (%)]	16 (34.0)	22 (53.7)

Reported missing data does not include patients lost to follow-up.

^a^Missing data: n = 1 (2.0%) baseline, n = 1 (2.0%) follow-up; ^b^Missing data: n = 0 (0.0%) baseline, n = 1 (2.0%) follow-up; ^c^Missing data: n = 0 (0.0%) baseline, n = 3 (7.0%) follow-up; ^d^BPI-ANCA positive: anti-BPI IgG > 10; missing data: n = 0 (0.0%) baseline, n = 3 (7.0%); ^e^Missing data: n = 0 (0.0%) baseline, n = 1 (2.0%) follow-up.

### Lung function

Forced expiratory volume in one second (FEV1) was measured by spirometry during the participants routine clinic visits following the American Thoracic Society (ATS) established guidelines for lung function studies^10^. An office spirometry machine was used for the study, and the results were expressed using proportion of predicted values obtained using computer calculated reference values based on the patients’ age, sex and height. The FEV1 was repeated at the 1 year follow up and compared to the initial result. The number of pulmonary exacerbations and CFTR modulator drug use during the year prior to each encounter were noted and recorded.

### Detection of antibodies against BPI

After informed consent was obtained from the subjects, about 5 mL of blood were drawn for BPI-ANCA analysis. The samples were transported to the laboratory and centrifuged at 2500 rpm for 20 min to separate the plasma from red blood cells, white blood cells, and platelets. After centrifugation the top layer of the sample, which contains the plasma, was collected, aliquoted, and stored at − 20 °C until use.

To quantify the amount of bactericidal/permeability protein (BPI) in plasma collected from patients or healthy volunteers, the anti-BPI ELISA test system (Orgentec) was used. The assay was performed according to the manufacturer’s instructions. Samples were diluted 1:100 and added to the wells of the plate. The samples were incubated at room temperature for 30 min and then washed three times with a wash buffer containing Tris, detergent, and sodium azide. Next the enzyme conjugate, which contained horseradish peroxidase (HRP) labeled, anti-human IgG antibodies, was added to the wells, and the wells were incubated for 15 min at room temperature. After washing with the same wash buffer, TMB (3,3′,5,5′-tetramethylbenzidine) substrate was added to the wells for 15 min to react with HRP and allow for a measurable color change. After washing, the reaction was stopped using a stop solution which contains acid. The optical density was measured at 450 nm (reference 650 nm) using an Infinite M100 PRO Quadruple monochromator microplate reader (Tecan, Switzerland).

### Variables

#### Outcomes

BPI-ANCA was treated as a dichotomous variable with anti-BPI IgG > 10 U/mL indicative of a positive result based on the manufacturer’s recommendation. The anti-BPI IgG results were obtained using ELISA assay system (Orgentec) and following the manufacturer’s system. The patients’ FEV1 percent predicted (FEV1%) was used as a measure of pulmonary function. The FEV1 percent predicted was part of the patient’s routine care and performed with spirometry machine following ATS guideline for spirometry interpretation. The number of times a patient was prescribed antibiotics for pulmonary exacerbation in the year prior to testing was recorded as a secondary measure of pulmonary function. This information was abstracted from each patient’s medical record at baseline and again at annual follow-up.

#### Predictors and other variables

In addition to serving as an outcome variable, anti-BPI IgG was also our primary predictor when modeling the relationship between BPI-ANCA and pulmonary function. It was included as a continuous variable unless stated otherwise. Other variables that might impact pulmonary function included age (continuous), colonization with *Pseudomonas aeruginosa* (yes/no), use of antibiotics and/or a CFTR modulator at time of testing (yes/no). Bacterial colonization was defined as re-isolation of the same bacterium in culture results after appropriate anti-microbial treatment without evidence of ongoing clinical signs and symptoms of infection.

The name of the antibiotic and modulator used was recorded at each time point.

Demographic and medical history data for our patients were recorded from the medical record, including sex (male/female), prior medical history of pancreatic insufficiency, asthma, gastroesophageal reflux disease, rheumatoid arthritis or diabetes (yes/no), and tobacco smoking status (active/not active).

### Statistical analysis

We conducted all statistical analyses in Stata/MP version 17.0 (StataCorps, LLC, College Station, Texas) and used listwise deletion for missing data^11^. In analyses in which we analyzed data as independent cross-sections, we did not remove patients who were lost to follow-up from the baseline analysis. We verified the healthy controls were negative for BPI-ANCA and removed them from the dataset prior to performing analyses.

We calculated descriptive statistics for continuous variables with mean/standard deviation or median/ interquartile range and number/percentage for categorical variables. To determine prevalence of BPI-ANCA, we calculated the proportion of patients who were positive for BPI-ANCA at baseline with a 95 percent confidence interval (CI), then again at follow-up. We also calculated the percent of change in status between baseline and follow-up.

#### Crude analysis

We used the Wilcoxon rank-sum test to test the hypothesis that there is a difference in pulmonary function (measured as FEV1% predicted) given the status of the BPI-ANCA. We selected an α = 0.05 a priori as threshold for statistical significance and performed the test separately for baseline and follow-up.

#### Multivariable analysis

To acknowledge the complexity of treatment among patients with CF, we used the full set of longitudinal data to perform multivariable generalized least squares (GLS) regression with random effects to control for potential confounding. The analysis was performed in the context of repeated measures and accounted for the shared variance during estimation. We also verified that random effects model was appropriate using the Hausman test. Our dependent variable was FEV1% predicted for patients at both time periods, and our primary predictor was a categorical iteration of anti-BPI IgG. We used the categorical variable because the assumptions of the regression model were violated with the continuous version. We dichotomized BPI-ANCA status: anti-BPI IgG ≤ 10.0 (BPI-ANCA negative) and > 10.0 (BPI-ANCA positive). We hypothesized that age, the use of antibiotics at time of testing, *Pseudomonas aeruginosa* colonization, and the use of a CFTR modulator at time of testing may confound the relationship between anti-BPI IgG and FEV1% predicted. We included these variables in the multivariable model, along with interaction terms between BPI-ANCA status and CFTR modulator use. We estimated robust errors and examined the Wald statistic and R-squared statistics for model goodness-of-fit. Last, we examined the intraclass correlation coefficient to better interpret the variance found in the model (Table 3).

**Table 3:** Results of multivariable generalized least squares regression with random effects modeling the relationship between anti-BPI IgG and FEV1% predicted.

Predictor	Coefficient	Robust SE	P-value	95% CI
BPI-ANCA positive	2.577	4.547	0.571	− 6.334, 11.488
CFTR modulator use (yes)	7.402	4.245	0.081	− 0.914, 15.722
Age	− 0.817	0.297	0.006	− 1.399, 0.234
Antibiotic use (yes)	− 0.292	3.669	0.937	− 7.483, 6.899
*Pseudomonas* colonization	0.209	6.281	0.973	− 12.101, 12.520

SE: standard error; CI: confidence interval; coefficients are included as percentage.

The full model included age, antibiotic use at time of visit, *Pseudomonas aeruginosa* colonization, BPI-ANCA status (+/−), and CFTR modulator use.

#### Post-hoc analyses

We chose to further explore the relationship between BPI-ANCA and pulmonary function by categorizing FEV1% predicted as normal (≥ 80% predicted) and low (< 80% predicted). We estimated the risk difference and risk ratio of low FEV1% predicted given the presence of BPI-ANCA. We calculated these scores separately by cross-section and used the Fisher’s exact test to formally test for differences in outcomes across groups (α = 0.05).

We also explored the effect of CFTR modulator use on anti-BPI IgG expression. We used the k-sample equality-of-medians test to test the hypothesis that anti-BPI IgG score would be different among patients who did and did not use a CFTR modulator. We also evaluated the relationship between BPI-ANCA and the number of pulmonary exacerbations experienced prior to testing. Neither variable was normally distributed, prompting the use of the medians test. We performed these tests independently at baseline and follow-up and interpreted either the Pearson’s chi-square or Fisher’s exact test with α = 0.05 defined a priori. Interpretation was decided based on the number of observations per cell—those with fewer than five observations per cell were interpreted using Fisher’s exact.

## Results

### Participants

The study included 52 participants, 47 of whom received care at our institution for CF. Five patients were otherwise healthy controls and eight patients (17.0%) were either lost to follow-up or had incomplete follow-up data. Sample characteristics at baseline are detailed in Tables 1 and 2. More than half of the patients enrolled were female (57.4%) and the mean age was 18.8 years. Almost all patients had a history of pancreatic insufficiency, and more than half had a history of asthma. Less than a quarter of participants had a history of GERD, diabetes, or rheumatoid arthritis, and only one patient was an active smoker at time of enrollment.

### Clinical results

The IgG BPI-ANCA results of the five healthy volunteers were all negative (range 2.51 to 5.39). Two of the healthy volunteers were available to follow-up and the results were negative (2.71 and 2.76). The median score for anti-BPI IgG was 4.2 with an interquartile range (IQR) of 3.0 to 7.3 (Table 2), irrespective of age. This remained relatively constant and was similar at follow-up. Both measures included extreme outliers (range at baseline = 1.72–213.8, follow-up = 1.79–226.1). Quality checks were performed on the outliers to verify accuracy. Approximately three-quarters of participants were on antibiotics at the time of enrollment with trimethoprim-sulfamethoxazole (26.5%), levofloxacin (23.5%) and ciprofloxacin (11.8%) the most commonly used. Eighty percent of patients at follow-up were on antibiotics at time of testing with a wider distribution of antibiotics. Fewer patients were on levofloxacin (11.8%), and more patients were on aztreonam (11.8%), tobramycin (14.7%) and azithromycin (14.7%) than at baseline.

Some patients were also using CFTR modulators at the time of enrollment (34.0%). Twenty-five percent of patients on CFTR modulators at enrollment were on Trikafta while 31.2% were on Orkambi and 37.5% were on Symdeko. Only one patient was taking Kalydeko at enrollment. The distribution of medication changed significantly at follow-up. Sixteen (72.7%) of patients on a CFTR modulator were on elexacaftor-tezacaftor-ivacaft while 18.2% were on lumacaftor-ivacaftor, and only 2 patients were on tezacaftor/ivacaftor or ivacaftor, respectively.

### Prevalence of BPI-ANCA

The proportion of participants with CF that were positive for BPI-ANCA at baseline was 0.19 (95% CI = 0.10, 0.33). The proportion of participants with CF that were positive for BPI-ANCA at follow-up was 0.15 (95% CI = 0.07, 0.31). Of participants positive at baseline, three (33.3%) converted to negative at the follow-up, and one was lost to follow-up. Only one participant (2.6%) negative at baseline converted to positive at follow-up Figures 3 and 4.

### Association of BPI-ANCA with pulmonary function

The data were analyzed to determine if the pulmonary function, as measured by FEV1% predicted, is different in individuals positive for BPI-ANCA compared to those who are negative. The result of the Wilcoxon rank-sum tests showed there was not a statistically significant difference in the mean FEV1% predicted value between groups at both baseline (p = 0.38) and follow-up (p = 0.41).

Additionally, longitudinal GLS regression with random effects showed there was no statistically significant difference in FEV1% predicted value between participants who were and were not positive for BPI-ANCA after controlling for age, antibiotic use at time of visit, and *Pseudomonas aeruginosa* colonization (p = 0.571) (Table 3). The interaction between BPI-ANCA status and CFTR modulator use was also not statistically significant (p = 0.100).

The Wald chi-square statistic for the model was statistically significant (p = 0.045) suggesting the model was appropriately specified. Interestingly, intraclass correlation in the model was high (rho = 0.775) suggesting the majority of variability in the model was between participants rather than within participants across time. Last, the within subjects R-square was 0.14, indicating the variables selected for our model explained only 14% of the variance within participants.

**Figure 1:**
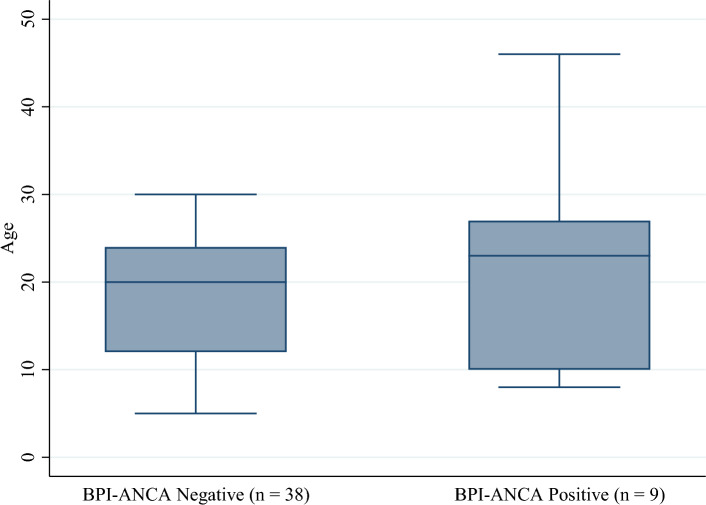
Distribution of age at baseline given BPI-ANCA status. Group differences tested via Independent Student’s t-test, α = 0.05; p = 0.261.

**Figure 2:**
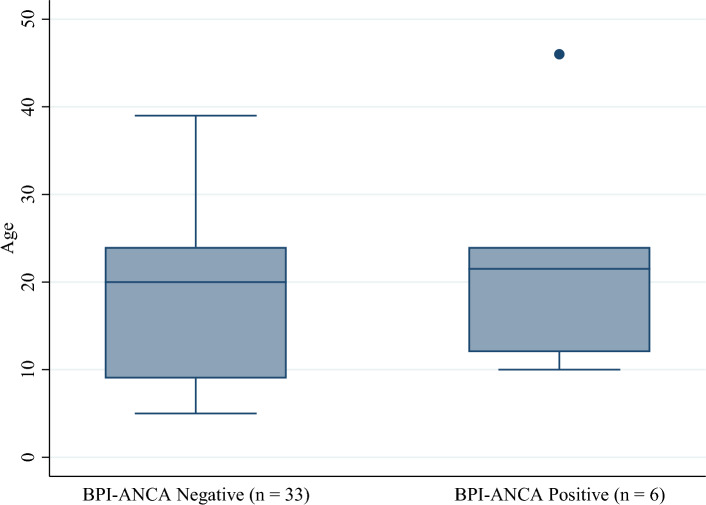
Distribution of age at follow-up given BPI-ANCA status. Group differences tested via Independent Student’s t-test, α = 0.05; p = 0.288.

**Figure 3:**
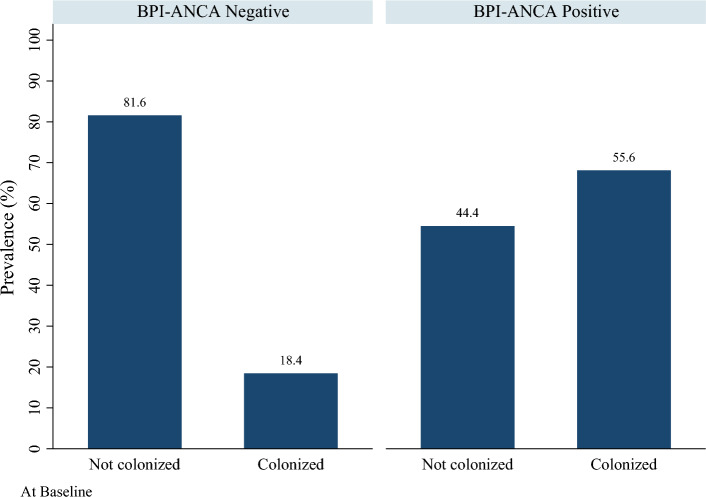
Prevalence of Pseudomonas colonization by BPI-ANCA status at baseline. Group differences tested via Fisher’s exact test, α = 0.05; p = 0.035.

**Figure 4:**
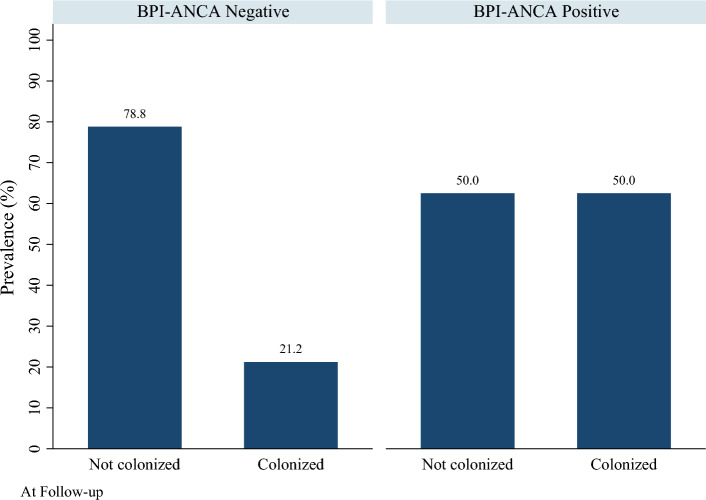
Prevalence of Pseudomonas colonization by BPI-ANCA status at follow-up. Group differences tested via Fisher’s exact test, α = 0.05; p = 0.163.

**Figure 5:**
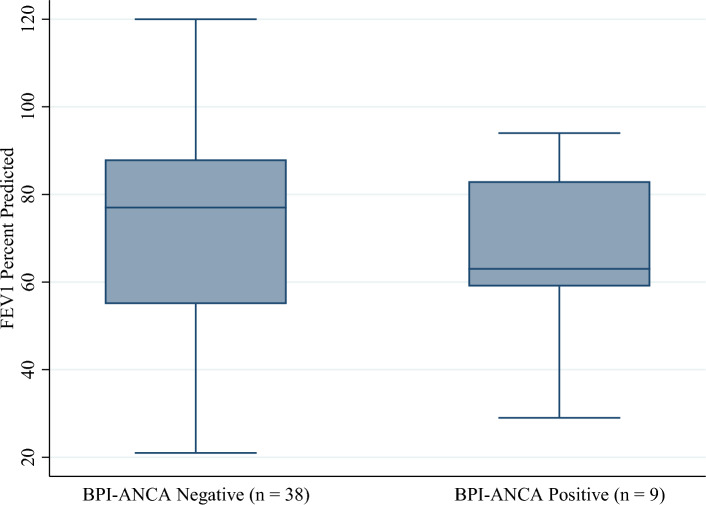
Distribution of FEV1 percent predicted by BPI-ANCA status at baseline. Group differences tested via Wilcoxon rank-sum test, α = 0.05; p = 0.383.

**Figure 6:**
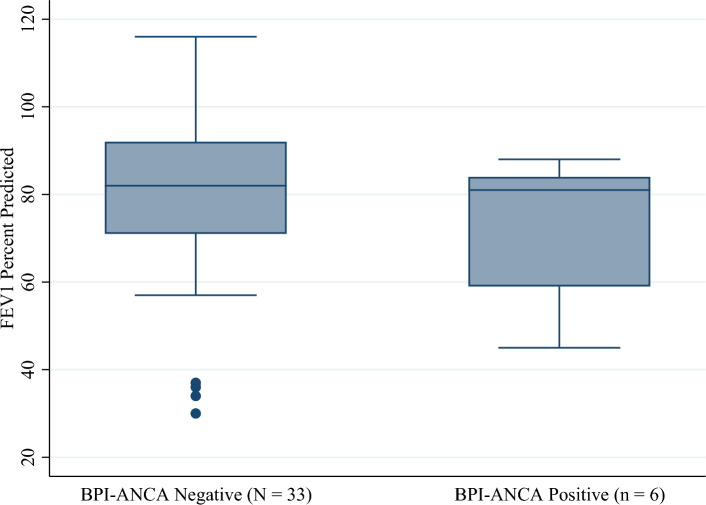
Distribution of FEV1 percent predicted by BPI-ANCA status at follow-up. Group differences tested via Wilcoxon rank-sum test, α = 0.05; p = 0.411.

Contrary to other studies, our study did not show statistically significant difference in FEV1 and pulmonary exacerbation in CF patients with BPI-ANCA positivity compared to those who tested negative for BPI-ANCA. There was not a statistically significant difference in mean age between groups at either baseline or follow-up (Figs. 1 and 2). However, there was a statistically significant difference in the proportion of patients who were colonized with *Pseudomonas* at both baseline and follow-up (Figs. 3 and 4). Greater than 50% of patients positive for BPI-ANCA were colonized with *Pseudomonas* at baseline compared to only 18.4% in the BPI-ANCA negative group. This difference did not hold through follow-up, potentially due to lost patient during follow-up. Last FEV1 values were lower in patients with BPI-ANCA, but the difference was not statistically significant (Figs. 5 and 6). Finally, while there was no difference in median score of anti-BPI IgG with CFTR modulator use at baseline, median score of anti-BPI IgG at follow up was higher for patients not on CFTR modulator versus those on CFTR modulator. This is statistically significant and may reflect a difference in CFTR modulator type at baseline versus follow up.

### Post hoc analyses

Upon exploring the crude relationship between BPI-ANCA positivity and low pulmonary function (FEV1% predicted < 80%), there was not a statistically significant difference in the risk of low pulmonary function between groups at baseline (Fisher’s exact p = 0.713). The risk difference between patients with BPI-ANCA and those without was 0.11 (95% CI = − 0.23, 0.46). A similar relationship was present at follow-up (Fisher’s exact p = 1.00) with a risk difference of − 0.06 (95% CI = − 0.47, 0.35). In addition, using the k-sample test, there was not a statistically significant difference in the median number of pulmonary exacerbations between patients with BPI-ANCA and those without. This was true at baseline (p = 0.28) and at follow-up (p = 1.00).

Last, we found there was not a statistically significant difference (p = 0.13) in the median score of anti-BPI IgG given CFTR modulator use at baseline. However, we found there was a statistically significant difference (p = 0.02) in the median score of anti-BPI IgG given CFTR use at follow-up. The median anti-BPI IgG for patients not on the CFTR modulator was 4.55 (IQR = 2.97, 7.34) while the median for patients on the CFTR modulator was 2.97 (IQR = 2.97, 3.68).

## Discussion

The increase in BPI-ANCA in patients with *Pseudomonas aeruginosa* and other inflammatory conditions, such as vasculitis, cystic fibrosis, inflammatory bowel disease and others, has been increasingly reported. Most of the studies on BPI-ANCA done outside the United States suggest inverse correlation between BPI-ANCA and pulmonary function in patients with CF. An increase in BPI-ANCA is also associated with pulmonary exacerbations in this patient population.

A recent meta-analysis showed the prevalence of BPI-ANCA in patients with CF varied from 17.9 to 83% with a pooled prevalence of nearly 50%^7^. In our study, the prevalence of BPI-ANCA in CF patients were 19% and 15% at initial and annual follow-up visit, respectively. Three of those who were positive at the initial visit converted back to negative at their 1 year follow up visit, indicating dynamic nature of this autoantibody. A plausible explanation for the low prevalence of BPI-ANCA noted in our study as compared others may be the fact that majority of our patients are on CFTR modulators which were not widely available a decade ago when the other studies were done. Studies on CFTR modulators in patients with CF have shown a pulmonary protective effect based on stable FEV1s and decreased pulmonary exacerbations^12,13^.

## Limitations

This study has a number of limitations. First, there may be individual variability in the production of measurable antibody titers which may depend on the disease process. Also, this study measured only IgG levels which reflect the chronicity of a disease process. IgA and IgM may develop early in an autoimmune process, and measuring these antibodies might increase the number of positive antibody tests as reported in other studies. The majority of studies on BPI-ANCA autoantibodies that showed strong clinical correlation was done using IgA BPI-ANCA. We plan on repeating similar study in the future focusing on IgA related auto-antibodies in patients with CF. Second, the number of patients recruited in this study may have been inadequate to show statistically significant associations between antibody levels and clinical factors. Also, there may be unknown geographical and environmental effects (possibly related to genetic profiles) in the development of autoimmunity. Additional studies need to be done in other CF centers in the United States. Third, antibiotic use and CTFR modulators (especially elexacaftor-tezacaftor-ivacaft) may have affected the production and persistence of these autoantibodies in CF patients, but our data set cannot clarify this possibility. The majority of our patients 12 years and older are were already on elexacaftor-tezacaftor-ivacaft during their annual follow-up visit. Future studies might focus on understanding the role of CTFR modulators on auto-antibodies.

## Conclusion

This study was designed to determine the prevalence and clinical associations of BPI-ANCA IgG levels in CF patients in United States. As compared to previously published studies, our results did not show any statistically significant difference in age, CFTR modulator use, or FEV1 in patients classified by BPI-ANCA status. However, there was a difference in *Pseudomonas* colonization at baseline.

### Supplementary Information

Supplementary Information.

## Data Availability

All data generated or analyzed during this study are included in this published article (and its supplementary information files).
