# Supplementary material for: Clinical significance of BPI-ANCA in patients with cystic fibrosis: a single center prospective study
Source: Sci Rep. 2023 Oct 24;13:18138. doi: 10.1038/s41598-023-45273-2 (PMC10598027; doi:10.1038/s41598-023-45273-2)
Supplement: Supplementary file 1 — Supplementary Information. [file 41598_2023_45273_MOESM1_ESM.pdf]

id	group	Age	Gender	pulm_exacerbation_baseline	pulm_exacerbation_second	other_medhist_pancreatic_insufficiency	other_medhist_asthma	other_medhist_GERD	diabetes	organ_transplant_yn	rheumatoid_arthritis	smoker	antibiotic_visit1	antibiotic_visit1_name1	antibiotic_visit1_name2	antibiotic_visit1_name3	antibiotic_visit2	antibiotic_visit2_name1	antibiotic_visit2_name2	antibiotic_visit2_name3	*Pseudomonas* colonization	FEV1_baseline	FEV1_baseline_percentage	FEV1_second	FEV1_second_percentage	BPI_IgG_baseline	BPI_IgG_second	CFTR_modulator	CFTR_modulator_name	CFTR modulator at start enrollment and start	CFTR modulator at end of enrollment and start	loss_followup	
1	1	6	1	4	5	1	0	0	0	0	0	0	1	1	14	0	1	1	14	0	0	0.89	55	1.59	98	3.46	2.97	1	2	1	1	0	
2	1	19	1	3	.	1	0	0	0	0	1	0	1	2	0	0	0	.	.	.	0	1.30	44	.	.	3.83	.	0	0	0	0	1	
3	1	15	0	7	8	1	1	0	0	0	0	0	0	.	.	.	1	7	8	0	1	2.62	73	3.70	95	7.37	4.49	1	1	0	0	0	
4	1	22	0	3	2	1	1	0	0	0	0	0	1	4	0	0	0	.	.	.	0	3.61	88	3.27	71	5.38	2.97	1	1	0	0	0	
5	1	28	0	8	8	1	0	1	0	0	0	0	1	5	0	0	1	7	0	0	0	3.25	65	3.47	71	3.82	2.93	1	3	1	1	0	
6	1	15	1	8	12	1	1	0	1	0	0	0	1	14	0	0	1	15	0	0	1	1.31	46	1.82	61	4.74	2.76	1	1	1	1	0	
7	1	25	0	3	6	1	1	0	0	0	0	0	1	2	0	0	1	2	0	0	0	3.95	89	3.76	85	4.70	2.79	1	1	0	0	0	
8	1	20	1	2	4	1	0	0	0	0	0	0	1	14	0	0	1	14	0	0	0	3.12	89	3.12	90	2.16	4.48	1	1	0	0	0	
9	1	10	1	5	5	1	1	1	0	0	0	0	1	6	14	0	1	14	0	0	0	2.04	97	2.26	97	4.39	2.71	0	0	0	0	0	
10	1	9	1	4	4	1	0	0	0	0	0	0	1	14	0	0	1	2	0	0	0	1.33	85	1.55	84	2.16	4.48	0	0	0	0	0	
11	0	30	0	0	0	0	0	0	0	0	0	0	0	.	.	.	0	.	.	.	.	.	.	.	.	4.39	.	0	0	0	0	0	
12	0	30	0	0	0	0	0	0	0	0	0	0	0	.	.	.	0	.	.	.	.	.	.	.	.	3.68	.	0	0	0	0	0	
13	1	24	1	2	1	1	0	0	0	0	0	0	1	8	14	0	1	8	0	0	1	1.85	41	3.67	81	72.07	17.38	1	1	1	1	0	
14	1	23	1	5	11	1	1	1	0	0	0	0	0	.	.	.	1	7	6	.	1	2.44	77	2.56	81	32.48	20.93	1	1	0	0	0	
15	0	26	0	0	0	0	0	0	0	0	0	0	0	.	.	.	0	.	.	.	.	.	.	.	.	2.51	.	0	0	0	0	0	
16	0	26	0	0	0	0	0	0	0	0	0	0	0	.	.	.	0	.	.	.	.	.	.	.	.	5.39	.	0	0	0	0	0	
17	1	21	0	3	4	0	1	0	1	0	0	0	1	14	0	0	1	16	0	0	0	2.15	70	2.48	78	2.74	3.63	1	3	0	1	0	
18	1	46	1	3	4	1	1	0	1	0	1	0	1	10	0	0	1	8	0	0	0	0.82	29	1.25	45	37.75	44.54	1	1	1	1	0	
19	1	30	0	2	3	1	0	0	1	0	0	0	1	2	8	0	1	2	0	0	0	3.14	77	2.93	72	5.36	4.55	1	1	0	0	0	
20	1	23	1	5	3	1	1	0	0	0	0	0	1	10	0	0	1	14	7	0	1	2.14	67	2.97	81	4.38	5.17	1	1	0	0	0	
21	1	22	0	2	6	1	1	0	0	0	0	0	1	9	0	0	1	8	7	0	0	0.86	21	1.39	34	2.09	2.72	1	1	1	1	0	
22	0	33	1	0	0	0	0	0	0	0	0	0	0	.	.	.	.	.	.	.	.	.	.	.	.	3.24	.	0	0	0	0	0	
23	1	17	1	10	9	1	0	0	0	0	0	0	1	10	2	0	1	11	.	.	0	1.85	53	1.64	57	2.95	2.97	1	1	1	1	0	
24	1	8	1	2	4	1	0	0	0	0	0	0	0	.	.	.	1	2	0	0	0	1.43	94	1.41	78	30.91	7.70	0	0	0	0	0	
25	1	20	0	4	3	1	0	0	0	0	0	0	1	2	0	0	1	2	0	0	0	4.53	98	4.41	95	5.48	3.68	1	4	1	1	0	
26	1	7	0	4	4	1	1	0	0	0	0	0	1	2	0	0	0	0	0	0	0	1.27	90	1.39	89	3.02	1.79	1	2	1	1	0	
27	1	25	1	2	3	1	1	1	0	0	1	0	1	4	0	0	0	0	0	0	0	2.78	103	2.99	111	3.58	3.44	1	1	0	1	0	
28	1	27	1	3	.	1	0	0	0	0	0	0	1	2	10	0	0	0	0	0	1	2.61	86	.	.	12.29	.	1	1	0	0	1	
29	1	29	1	5	4	1	1	0	0	0	0	0	1	14	0	0	1	6	8	2	0	0.42	33	0.60	37	8.67	6.27	0	0	0	0	0	
30	1	39	1	8	4	1	1	0	1	0	0	0	1	14	2	0	1	17	6	11	1	2.11	61	1.01	36	13.75	4.56	1	1	1	0	0	
31	1	24	1	4	4	1	1	0	0	0	0	0	1	11	12	8	1	6	0	0	1	2.35	68	3.19	93	4.15	2.28	1	1	1	1	0	
32	1	20	0	5	5	1	1	0	1	0	0	0	0	.	.	.	1	1	8	0	0	3.53	78	3.66	82	3.82	2.17	1	1	0	1	0	
33	1	12	1	3	4	1	1	0	0	0	0	0	0	.	.	.	1	8	7	0	1	1.36	63	2.10	84	213.76	142.67	1	1	0	1	0	
34	1	16	0	3	5	1	1	0	0	0	0	0	1	9	0	0	1	15	7	0	0	1.10	31	1.20	30	3.16	1.86	0	0	0	0	0	
35	1	24	1	4	4	1	0	0	0	0	1	0	0	.	.	.	0	.	.	.	0	3.51	77	2.71	86	6.40	2.72	1	1	0	1	0	
36	1	12	0	2	5	0	0	0	0	0	0	0	1	14	18	0	1	6	0	0	0	0.91	46	1.55	71	8.62	6.9	1	1	0	0	0	
37	1	7	0	3	4	1	1	0	0	0	0	0	1	8	0	0	0	0	0	0	0	1.16	86	1.32	87	4.15	.	0	0	0	0	0	useable
38	1	5	1	6	2	1	1	0	0	0	0	0	0	.	.	.	0	.	.	.	0	1.11	120	1.23	116	2.79	2.04	1	2	1	1	0	
39	1	10	1	1	2	1	1	0	0	0	0	0	1	8	7	0	1	14	0	0	0	0.83	59	0.92	59	202.18	226.05	0	0	0	0	0	
40	1	5	0	1	3	1	0	0	0	0	0	0	0	.	.	.	1	1	0	0	0	1.25	69	1.15	81	4.08	3.12	1	2	1	1	0	
41	1	24	1	3	1	1	1	0	1	0	1	0	1	7	13	0	1	6	0	0	1	1.12	40	1.27	45	2.75	.	1	1	0	0	0	useable
42	1	8	0	3	2	1	1	0	0	0	0	0	1	18	0	0	1	4	0	0	0	1.45	83	1.57	82	118.30	9.36	0	0	0	0	0	
43	1	21	0	1	.	1	0	0	0	0	0	0	1	2	0	0	.	.	.	.	0	1.16	24	.	.	3.58	.	1	1	0	0	1	
44	1	5	1	3	4	1	1	0	0	0	0	0	1	14	0	0	1	7	0	0	1	0.79	85	0.81	73	2.72	2.05	0	0	0	0	0	
45	1	20	1	1	5	1	0	0	1	0	1	0	1	2	0	0	1	7	8	0	0	2.84	77	3.22	88	4.03	10.38	1	1	0	1	0	
46	1	19	1	3	4	1	1	0	0	0	0	0	1	2	8	0	1	2	8	0	0	3.27	95	3.44	101	2.99	2.21	1	1	1	1	0	
47	1	13	0	2	.	1	1	0	0	0	0	0	0	.	.	.	.	.	.	.	0	2.42	86	.	.	4.14	.	0	0	0	0	1	
48	1	28	0	2	.	1	0	1	0	0	0	0	0	.	.	.	.	.	.	.	0	3.36	84	.	.	1.72	.	0	0	0	0	1	
49	1	30	1	4	6	1	1	0	1	0	0	0	1	10	12	0	1	8	7	6	1	3.01	82	3.41	91	5.87	3.64	1	1	1	1	0	
50	1	26	1	1	.	1	0	1	1	0	0	0	0	.	.	.	.	.	.	.	0	.	.	.	.	2.89	7.34	0	0	0	0	0	
51	1	6	0	0	0	1	1	0	0	0	0	0	0	.	.	.	0	.	.	.	0	1.25	93	1.40	86	5.49	5.41	1	1	0	0	0	
52	1	17	0	4	2	1	1	1	0	0	0	1	0	.	.	.	1	3	0	0	0	2.84	77	2.57	66	2.38	.	1	1	1	1	0	useable

Supplemental 1. Data collection sheet

Patient Sample	Date of Blood Draw	Average	Concentration (U/mL)	Notes:
31	4/4/2019	0.26	4.15	
32	4/18/2019	0.24	3.82	
33	4/16/2019	2.76	213.76	High
34	5/23/2019	0.17	3.16	
35	5/30/2019	0.40	6.40	
36	7/19/2019	0.49	8.62	
37	9/8/2019	0.26	4.15	
38	9/19/2019	0.09	2.79	
39	10/11/2019	2.68	202.18	High
40	11/26/2019	0.26	4.08	
41	12/19/2019	0.08	2.75	
1 Final	12/3/2019	0.14	2.97	
3 Final	10/10/2019	0.29	4.49	
5 Final	11/12/2019	0.13	2.93	
7 Final	11/26/2019	0.09	2.79	
8 Final (HV 1)	12/4/2019	0.06	2.71	Healthy Volunteer
14 Final	11/12/2019	0.84	20.93	First draw was previously high
23	7/3/2019	0.13	2.95	
17	6/20/2019	0.08	2.74	
21 Final	1/9/2020	0.07	2.72	
43	1/10/2020	0.21	3.58	
4 Final	1/10/2020	0.14	2.97	
42	1/8/2020	2.05	118.30	High
10 Final	1/14/2020	0.29	4.48	
6 Final	1/14/2020	0.08	2.76	
44	1/16/2020	0.12	2.86	
9 Final (HV 2)	1/14/2020	0.09	2.76	Healthy Volunteer
25 Final	1/28/2020	0.22	3.68	
13 Final	1/29/2020	0.76	17.38	First draw was previously high

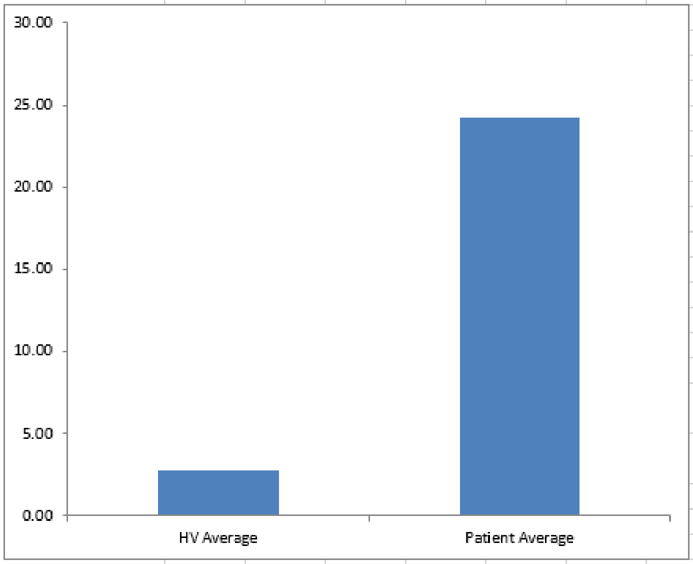

Supplemental 2. Raw Data collection

Dual wavelength measurement with reference wavelength

0.0487	0.0653	0.0639	0.2247	0.0632	0.0692	0.0758	0.0671	0.0619	0.1852	0.0649	0.062799998
0.05	0.0534	0.0461	0.1873	0.044	0.053	0.0624	0.055	0.0405	0.1698	0.0464	0.038699999
0.0615	0.0685	0.0615	0.1852	0.0439	0.0526	0.0606	0.0494	0.0416	0.1489	0.0499	0.0447
0.0782	0.0423	0.0675	0.0428	0.0407	0.0384	0.0735	0.0422	0.0397	0.0392	0.0458	0.044500001
0.1153	0.0582	0.0642	0.0606	0.0566	0.0564	0.0845	0.0586	0.0555	0.0541	0.0591	0.055599999
0.1346	0.0425	0.0487	0.0409	0.0393	0.038	0.0705	0.0438	0.0377	0.0366	0.0445	0.042199999
0.0858	0.1647	0.1507	0.1515	0.0525	0.0507	0.0586	0.0412	0.0419	0.0416	0.0668	0.066699997
0.041	0.0411	0.0437	0.0446	0.0439	0.0411	0.0396	0.0436	0.0415	0.0504	0.0368	0.073600002

Calculated difference between measurement and reference measurement

	0.0114	0.2797	0.2517	3.3518	0.2241	0.3965	0.4668	0.2622	0.0938	3.1055	0.2771	0.0858
	0.3063	0.2489	0.2226	2.7975	0.1416	0.3884	0.5228	0.2596	0.0823	2.8354	0.2286	0.069200002
	0.5111	0.2646	0.2388	2.1323	0.1326	0.4131	0.4944	0.273	0.1081	2.1136	0.2723	0.092900001
	0.9151	0.1396	0.3317	0.1345	0.1009	0.0539	0.8775	0.1352	0.0873	0.0695	0.2231	0.136199996
	1.3973	0.1405	0.2709	0.133	0.0931	0.0615	0.8312	0.1007	0.0806	0.0693	0.2153	0.152400002
	1.8622	0.1308	0.2667	0.1198	0.0902	0.0578	0.8071	0.1635	0.0695	0.0592	0.2051	0.1228
	0.9509	2.0658	2.0633	2.0303	0.3054	0.2898	0.2719	0.0854	0.0809	0.0868	0.7316	0.699100018
	0.1142	0.1078	0.1014	0.1367	0.0997	0.0925	0.0653	0.1807	0.1309	0.3605	0.0265	0.839299977

End Time 2/24/2020 2:31:19 PM

Calibrato	Concentration	
A	0	0.0114
B	6.3	0.3063
C	12.5	0.5111
D	25	0.9151
E	50	1.3973
F	100	1.8622

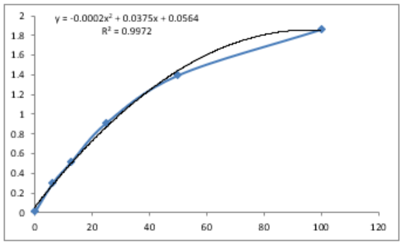

A	0.0114	0
B	0.3063	6.3
C	0.5111	12.5
D	0.9151	25
E	1.3973	50
F	1.8622	100

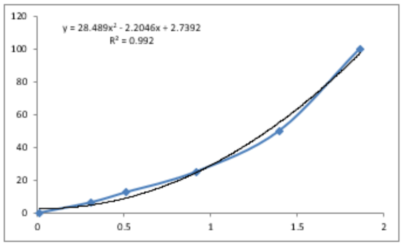

Group	Average	Concentration
31	0.26	4.15
32	0.24	3.82
33	2.76	213.76
34	0.17	3.16
35	0.40	6.40
36	0.49	8.62
37	0.26	4.15
38	0.09	2.79
39	2.68	202.18
40	0.26	4.08
41	0.08	2.75
1 Final	0.14	2.97
3 Final	0.29	4.49
5 Final	0.13	2.93
7 Final	0.09	2.79
8 Final (HV 1)	0.06	2.71
14 Final	0.84	20.93
23	0.13	2.95
17	0.08	2.74
21 Final	0.07	2.72
43	0.21	3.58
4 Final	0.14	2.97
42	2.05	118.30
10 Final	0.29	4.48
6 Final	0.08	2.76
44	0.12	2.86
9 Final (HV 2)	0.09	2.76
25 Final	0.22	3.68
13 Final	0.76	17.38

+ Control	26.403	
- Control	2.859	
		Standard Deviation
HV Average	2.73	0.03746374
Patient Average	24.24	57.44200996

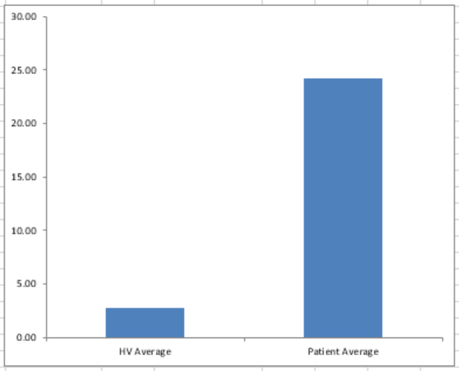

Supplemental 3. Raw Result

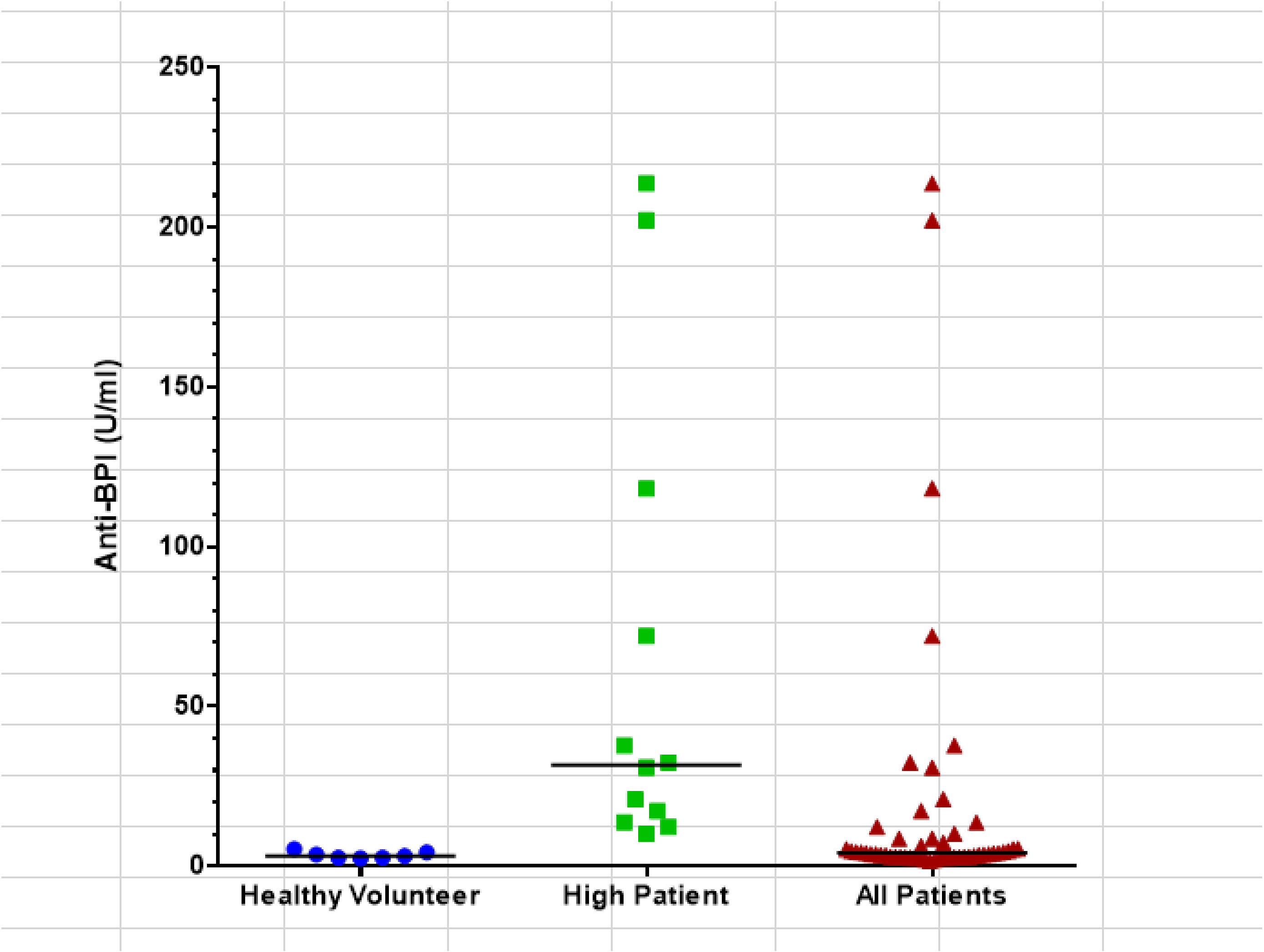

Supplemental 4. BPI-ANCA raw result summary
